# Development of a Nomogram With Alternative Splicing Signatures for Predicting the Prognosis of Glioblastoma: A Study Based on Large-Scale Sequencing Data

**DOI:** 10.3389/fonc.2020.01257

**Published:** 2020-07-22

**Authors:** Zihao Wang, Lu Gao, Xiaopeng Guo, Chenzhe Feng, Wei Lian, Kan Deng, Bing Xing

**Affiliations:** ^1^Department of Neurosurgery, Peking Union Medical College Hospital, Chinese Academy of Medical Sciences and Peking Union Medical College, Beijing, China; ^2^Chinese Pituitary Adenoma Cooperative Group, China Pituitary Disease Registry Center, Beijing, China

**Keywords:** glioblastoma, alternative splicing, splicing factor, prognostic model, TCGA

## Abstract

**Purpose:** Alternative splicing (AS) was reported to play a vital role in development and progression of glioblastoma (GBM), the most common and fatal brain tumor. Systematic analysis of survival-associated AS event profiles and prognostic prediction model based on multiple AS events in GBM was needed.

**Methods:** Genome-wide AS and RNA sequencing profiles were generated in 152 patients with GBM in the cancer genome atlas (TCGA). Prognosis-associated AS events were screened by integrated Cox regression analysis to construct the prognostic risk score model in the training cohort (*n* = 101). The AS-based signature and clinicopathologic parameters were applied to construct a prognostic nomogram for 0.5-, 1-, and 3-year OS prediction. Finally, the regulatory networks between prognostic AS events and splicing factors (SFs) were constructed.

**Results:** A total of 1,598 prognosis-related AS events from 1,183 source genes were determined. Eight prognostic risk score model based on integrated AS events and 7 AS types were established, respectively. Concordance index (C-index) and receiver operating characteristic (ROC) curve analysis demonstrated powerful ability in distinguishing patients' outcomes. Only Alternate Donor site (AD) and Exon Skip (ES) signature out of the eight types of AS signature were identified as independent prognostic factors for GBM, which was validated in the internal validation cohort. The nomogram with age, new event, pharmaceutical therapy, radiation therapy, AD signature and ES signature were constructed, with C-index of 0.892 (95% CI, 0.853–0.931; *P* = 5.13 × 10^−15^). Calibration plots, ROC, and decision curve analysis suggested excellent predictive performance for the nomogram in both TCGA training cohort and validation cohort. Splicing network indicated distinguished correlations between prognostic AS events and SFs in GBM patients.

**Conclusions:** AS-based prediction model could serve as a promising prognostic predictor and potential therapeutic target for GBM, facilitating better treatment strategies in clinical practice.

## Introduction

Glioma is the most common primary tumor of the central nervous system, accounting for 40–50% of brain tumors in the United States from 2010 to 2014 (1). Glioblastoma (GBM), corresponding to WHO grade IV, is the most commonly occurring and most lethal type of glioma, generally exhibiting a 5-year overall survival (OS) rate of ~5% (1, 2). Despite remarkable advances in the development of managements for GBM, including surgery, chemotherapy, radiotherapy, targeted therapy, and immunotherapy, the optimal treatment strategy remains controversial (3). It has been reported in the literature that age, extent of resection, and various molecular alterations can serve as prognostic factors for GBM (4–6). Numerous clinical and molecular studies of GBM have been reported in recent years (4–6). However, the exact underlying mechanisms that contribute to its development, progression and recurrence have not been clearly elucidated. Hence, exploring the underlying molecular mechanisms and investigating prognostic biomarkers and predictors of therapeutic response are indispensable for the treatment of GBM patients.

Alternative splicing (AS) is a vital posttranscriptional process that modifies >95% of human genes by regulating the translation of mRNA isoforms and encoding splice variants in normal physiology (7, 8). Emerging evidence has demonstrated that aberrant AS events play a vital role in multiple cancers by promoting tumor cell proliferation, immune escape, metastasis, and drug resistance (9, 10). In addition, alterations or changes in the expression of splicing factors (SFs) could contribute to oncogenic AS events by activating oncogenes or cancer-related pathways and deactivating tumor suppressors (11). With the rapid development of large-scale genome-sequencing technologies, the roles of multiple oncogenic AS events in GBM have been investigated in recent years, including the associations between those AS events and GBM tumorigenesis, progression, recurrence and even treatment (12–14). Certain AS events and their cancer-specific mRNA isoforms can serve as promising therapeutic and prognostic biomarkers for GBM. However, systematic analysis of survival-associated AS event profiles and prognostic prediction models based on multiple AS events have not been realized in GBM before.

In this study, by performing a global expression profile assessment, we aimed to identify survival-related AS signatures that could serve as molecular biomarkers for subgroup classification, risk stratification, prognostication, and therapeutic targets in GBM. Moreover, we successfully established a novel, promising prognostic nomogram for GBM based on multiple AS signatures and clinicopathological factors, and we demonstrated its powerful predictive ability. Finally, the regulatory networks between prognostic AS events and SFs were constructed to investigate the underlying regulatory mechanisms.

## Materials and Methods

### Data Retrieval and Processing

**Figure 1:**
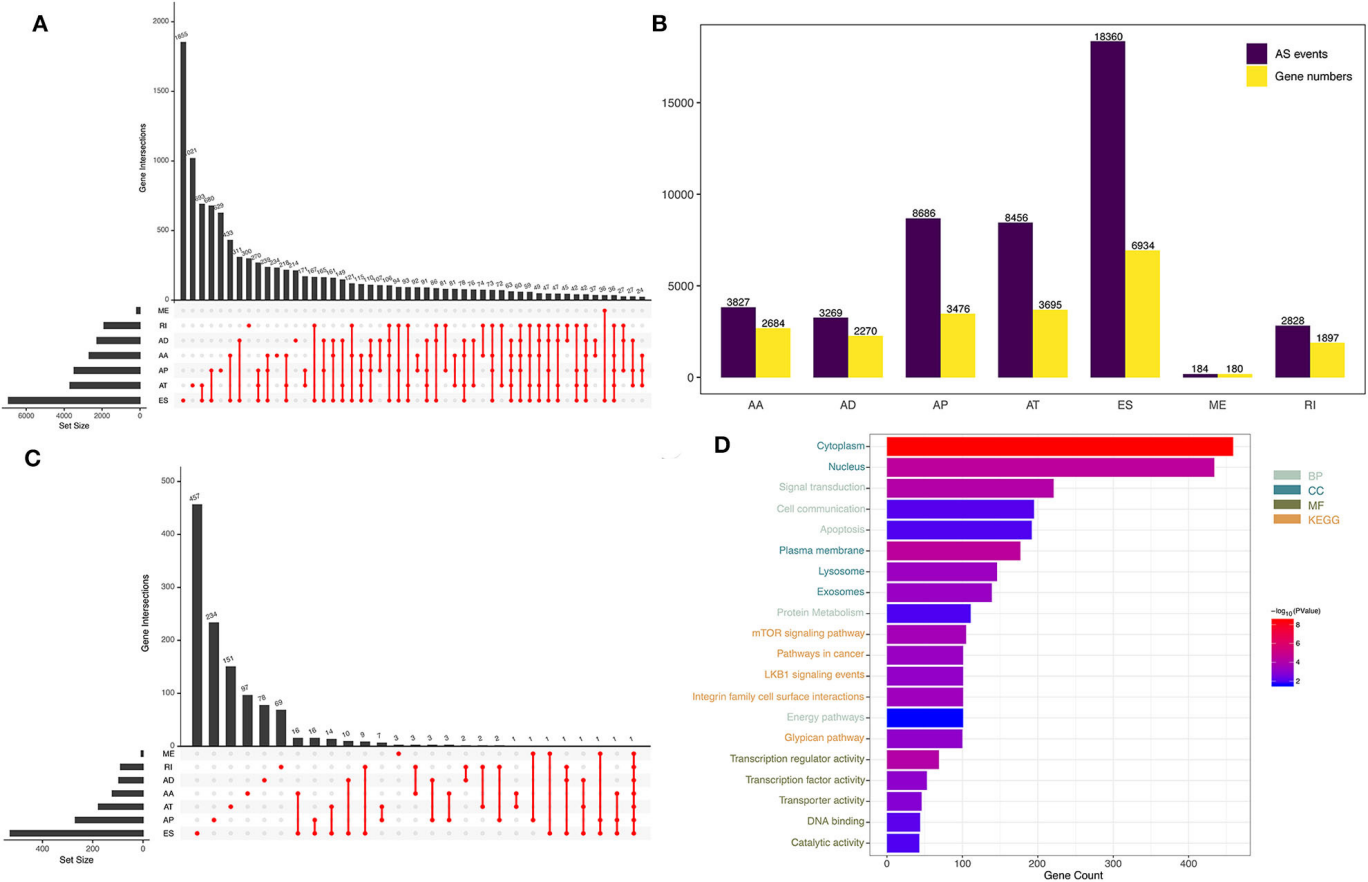
Overview of AS events in GBM patients. **(A)** UpSet plot of the seven types of AS events. **(B)** The source genes of the seven types of AS events. **(C)** The seven types of AS events and their association with survival, shown as an UpSet plot. **(D)** Functional enrichment analysis of the source genes of prognostic AS events.

The level 3 RNA sequencing data and clinical information of 156 GBM patients were downloaded from The Cancer Genome Atlas (TCGA, https://portal.gdc.cancer.gov/) database. Patients without prognostic information were excluded. AS events in GBM and their percent-splice-in (PSI) values were obtained from the TCGA SpliceSeq data portal (https://bioinformatics.mdanderson.org/TCGASpliceSeq/). PSI values are expressed on a scale from 0 to 1 and are commonly used to quantify AS events, providing an overview of the AS junction and the proportion of included exons from clinical samples (15). Seven types of AS events, namely, alternate acceptor site (AA), alternate donor site (AD), alternate promoter (AP), alternate terminator (AT), exon skip (ES), mutually exclusive exons (ME), and retained intron (RI) events, were quantified by PSI value (Figure 1A). Ultimately, a total of 152 GBM patients with TCGA SpliceSeq data, RNA sequencing data and clinical data were included our study. Four patients were excluded due to lack of prognostic information. Because the data were obtained from TCGA, our study did not require approval by an ethics committee.

### Construction of the Prognostic Risk Score Model Based on AS Events

First, all the 152 GBM patients were randomly divided into two groups on a ratio of 2:1, including training cohort (*n* = 101) and internal validation cohort (*n* = 51). To identify survival-associated AS events, we performed univariate Cox regression analysis to identify the associations between the PSI values of various AS events and patients' OS using the “survival” package (http://bioconductor.org/packages/survival/) in R 3.5.1 (16). The intersections between AS events as well as the quantitative analysis of these interactive sets were visualized as UpSet plots using the “UpSetR” package (17). Then, the Database for Annotation, Visualization and Integrated Discovery (DAVID, http://david.ncifcrf.gov/) was used to perform functional annotation and pathway enrichment analyses, including Gene Ontology (GO) and Kyoto Encyclopedia of Genes and Genomes (KEGG) pathway analysis, for the source genes of prognostic AS events (18–20). A *P*-value < 0.05 was considered statistically significant.

Then, prognostic AS events with a *P*-value < 0.05 in univariate Cox regression were further screened by least absolute shrinkage and selection operator (LASSO) regression. We adopted the optimal value of λ according to 10-fold cross-validation and the Akaike information criterion (AIC) and Bayesian information criterion (BIC) (21, 22). Finally, the optimal prognosis-related AS events were identified by multivariate Cox regression analysis to construct a prognostic risk score model for predicting OS. The prognostic risk score model was established with the following formula: risk score = PSI value of AS event_1_ × β_1_ + PSI value of AS event_2_ × β_2_ +…+ PSI value of AS event_n_ × β_n_, where β represents the regression coefficient calculated by the multivariate Cox regression model (23). Then, the prognostic risk score was generated for each patient. All GBM patients in the training set were divided into high-risk (high risk score) and low-risk (low risk score) groups using the median risk score as the cutoff. Kaplan-Meier (K-M) survival curve analysis using the “survival” package was performed to estimate the prognoses of patients with high and low risk scores, and the survival difference between the high-risk and low-risk groups was assessed by a two-sided log-rank test. The prognostic performance of the AS signature was evaluated by Harrell's concordance index (C-index) and time-dependent receiver operating characteristic (ROC) curve analysis of 0.5-, 1-, 2-, 3-, and 5-year survival with the “survcomp” (http://www.bioconductor.org/packages/survcomp/) and “survivalROC” (https://cran.r-project.org/web/packages/survivalROC/) R packages (24, 25). Both the C-index and the area under the curve (AUC) range from 0.5 to 1, with 1 indicating perfect discrimination and 0.5 indicating no discrimination. Finally, the prognostic model constructed by the TCGA training cohort were further validated by the internal validation cohort in a similar way. In addition, to determine whether the predictive power of the AS signature could be independent of other clinicopathological parameters, we performed univariate and multivariate Cox proportional hazards regression analyses in the training set and validation set.

### Construction and Validation of the Nomogram With AS Signatures

Demographics and clinical characteristics of the TCGA GBM patients in the training cohort and validation cohort were shown in Table 1. Clinicopathological parameters [including age, sex, new events, Karnofsky Performance Status (KPS) score, pharmaceutical therapy, radiation therapy, surgery, and IDH mutation status] and AS signatures (including the integrated AS signature as well as the AA, AD, AP, AT, ES, ME, and RI signatures), were entered into the univariate and multivariate Cox regression analysis. All the independent prognostic factors were determined to construct a prognostic nomogram to assess the probability of 0.5-, 1-, and 3-year OS for TCGA GBM patients using the “rms” R package (https://cran.r-project.org/web/packages/rms/) (26). The discrimination performance of the nomogram was quantitatively assessed by the C-index and the area under the ROC curve (24). Calibration plots were also used to graphically evaluate the discriminative ability of the nomogram (25). Additionally, decision curve analysis (DCA) was performed to determine the clinical usefulness of the prognostic nomogram by quantifying the net benefits at different threshold probabilities in GBM patients (27). The best prediction model is typically one that has a high net benefit within a suitable range of threshold probabilities. Finally, the prognostic nomogram was further validated by the internal validation cohort. All analyses were conducted using R version 3.5.1, and a *P*-value < 0.05 was considered statistically significant. Hazard ratios (HRs) and 95% confidence intervals (CIs) are reported if necessary.

**Table 1:** Demographics and clinical characteristics of the TCGA GBM patients in the training cohort (*n* = 101) and internal validation cohort (*n* = 51).

**Variables**	**Training set (*n* = 101)**	**Internal validation set (*n* = 51)**
Age (years)	61.1 ± 13.1	56.8 ± 14.3
Sex (Female/Male)	16/35	16/35
New event (None or NA/Yes)	47/54	18/33
KPS (<80/>= 80/NA)	21/50/30	11/32/8
Pharmaceutical therapy (CT only/CT + TMT/CT + HT/Others/No or NA)	41/16/11/2/31	22/11/9/3/6
Radiation therapy (No/Yes/NA)	18/76/7	4/47/0
Surgery (Biopsy only/Tumor resection)	11/90	6/45
IDH mutation status (Wildtype/Mutant)	97/4	47/4

*GBM, glioblastoma; NA, not available; KPS, Karnofsky performance score; CT, chemotherapy; TMT, targeted molecular therapy; HT, hormone therapy*.

*“New event” included progression and recurrence. “Others” in pharmaceutical therapy included CT + TMT + HT, CT + TMT + Immunotherapy, and CT + Immunotherapy*.

### Correlation Analysis and Regulatory Networks Between Prognostic AS Events and SFs

It has been reported that AS events in the tumor microenvironment can be modified or regulated by SFs (28). Hence, it is vital to explore the correlations between prognostic AS events and SFs in GBM. Correlation analyses between the PSI values of prognostic AS events and the expression levels of the corresponding SF genes were performed using Pearson's correlation test. A *P*-value < 0.001 combined with a correlation coefficient > 0.6 or < −0.6 was considered to indicate a significant correlation. Then, the regulatory networks between prognostic AS events and SFs were visualized using Cytoscape.

## Results

### AS Profiles in TCGA GBM Patients

By analyzing SpliceSeq data from 152 GBM patients, we obtained 45,610 mRNA splicing events in 21,136 source genes, including 3,827 AAs in 2,684 genes, 3,269 ADs in 2,270 genes, 8,686 APs in 3,476 genes, 8,456 ATs in 3,695 genes, 18,360 ESs in 6,934 genes, 184 MEs in 180 genes, and 2,828 RIs in 1,897 genes (Figure 1B). ES was the most common type of AS, accounting for 40% of all events, whereas ME was the least common.

### Prognostic AS Events and Functional Enrichment Analysis

Through univariate Cox regression analysis, we identified a total of 1,598 prognosis-related AS events in 1,183 source genes, as depicted in Figure 1C. GO analyses, including the biological process (BP), cellular component (CC) and molecular function (MF) categories, were performed on the source genes of prognostic AS events. In the BP category, there was significant gene enrichment in signal transduction, cell communication and apoptosis. In the CC category, genes were significantly enriched in the cytoplasm, nucleus and plasma membrane. In the MF category, genes were significantly enriched in transcription regulator activity, transcription factor activity and transporter activity. In addition, KEGG pathway analysis revealed significant gene enrichment in the mTOR signaling pathway, integrin family cell surface interactions and pathways involved in cancer (Figure 1D).

**Figure 2:**
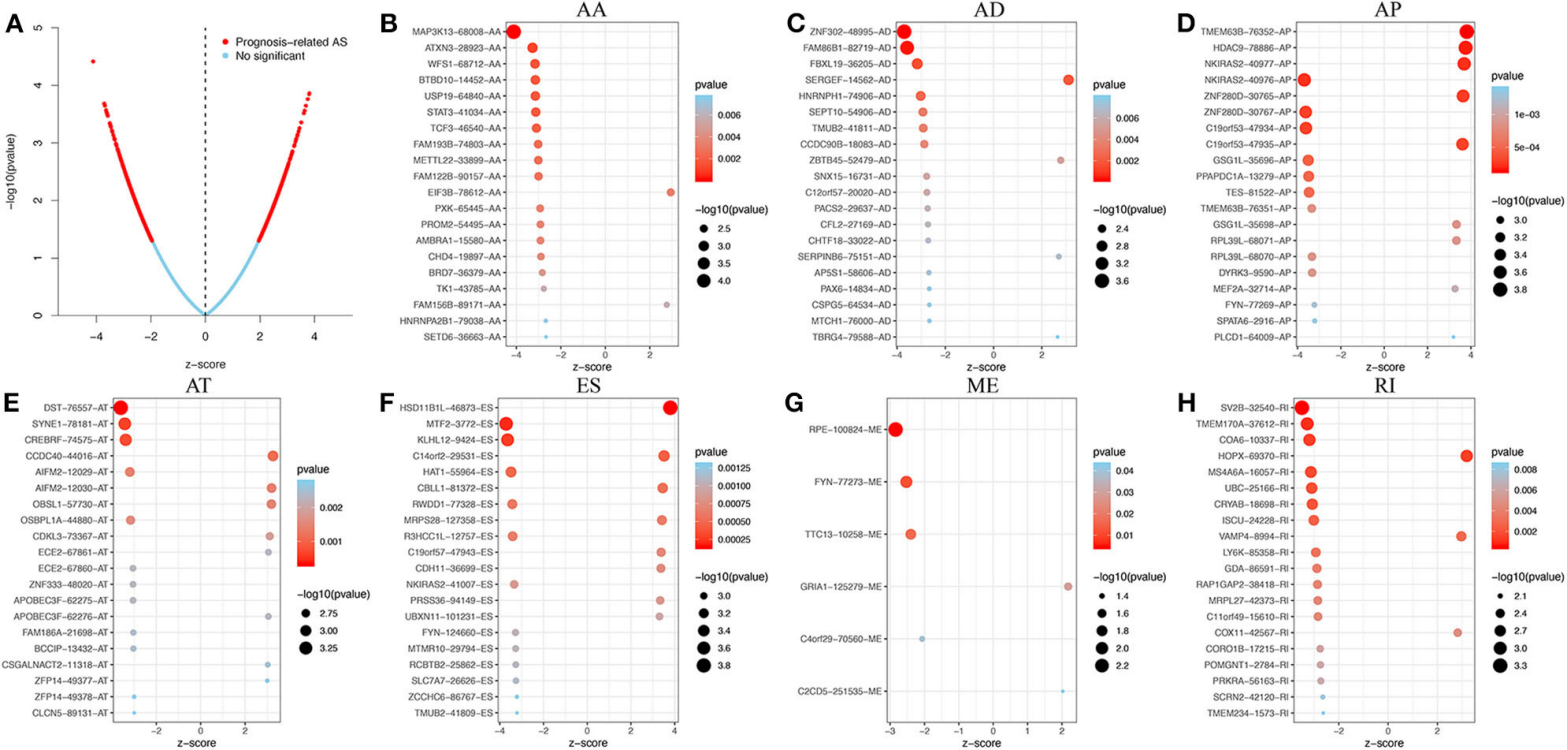
Prognosis-related AS events in GBM. **(A)** Volcano plot showing an overview of the prognosis-related AS events. **(B–H)** The top 20 prognosis-related AS events, illustrated by bubble plots, for AA, AD, AP, AT, ES, ME, and RI (z-score > 0, HR > 1; z-score <0, HR <1).

Using the HR or the z-score, each prognostic AS event was classified as a favorable (HR <1 or z-score <0) or unfavorable (HR > 1 or z-score > 0) prognostic factor. The top 20 most significant AS events of each of the seven types are presented graphically in Figure 2. Interestingly, most of the prognostic AS events were favorable prognostic factors (850 vs. 747).

### Construction of the AS-Based Prognostic Risk Score Models

**Figure 3:**
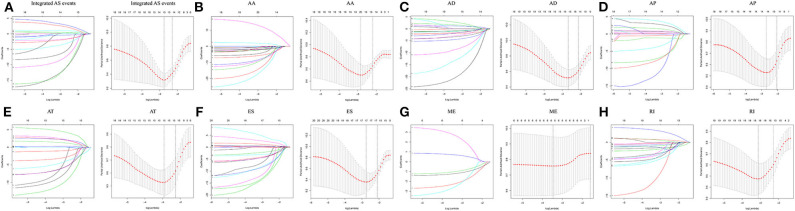
The prognosis-associated AS events were screened by LASSO regression analysis for all AS types combined and for each of the 7 AS types separately. Left panel **(A–H)** The coefficients are plotted against log(λ). A vertical line is shown at the value selected using 10-fold cross-validation, where the optimal λ results in ten features with nonzero coefficients. Right panel **(A–H)**: Optimal parameter (λ) selection in the LASSO model used 10-fold cross-validation via minimum criteria. The partial likelihood deviance (binomial deviance) curve is plotted vs. log(λ). Dotted vertical lines are shown at the optimal values selected using two different criteria: the minimum and 1 standard error of the minimum.

**Figure 4:**
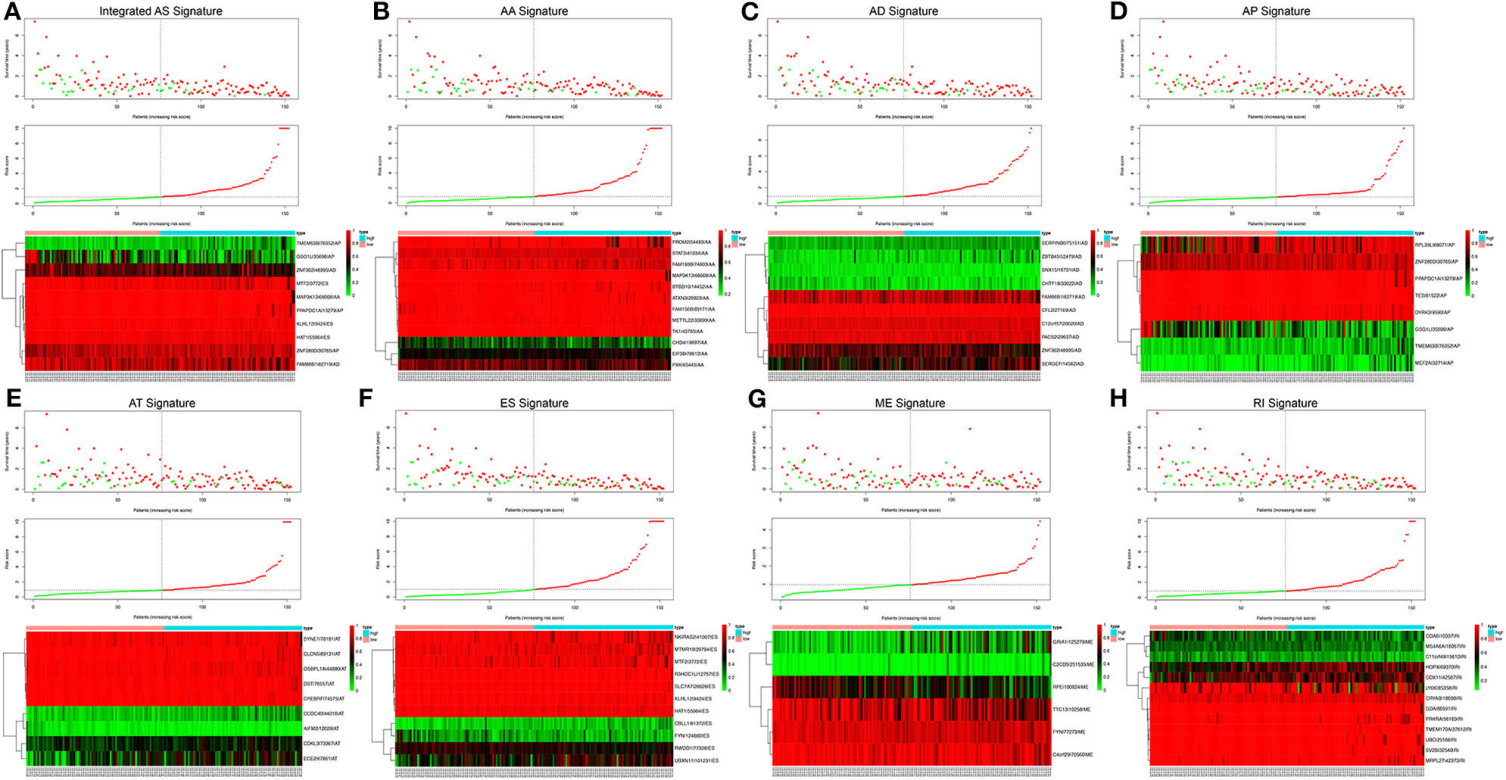
Risk score analysis of the integrated AS signature and the 7 type-specific AS signatures in GBM patients. Upper panel **(A–H)** Patient survival status and time distributed by risk score. Middle panel **(A–H)** Risk score curve of the AS signature. Bottom panel **(A–H)** Heatmaps of prognosis-related AS events. Colors ranging from green to red indicate expression levels ranging from low to high. The dotted line represents the individual inflection point of the risk score curve, by which the patients were categorized into low-risk and high-risk groups.

**Figure 5:**
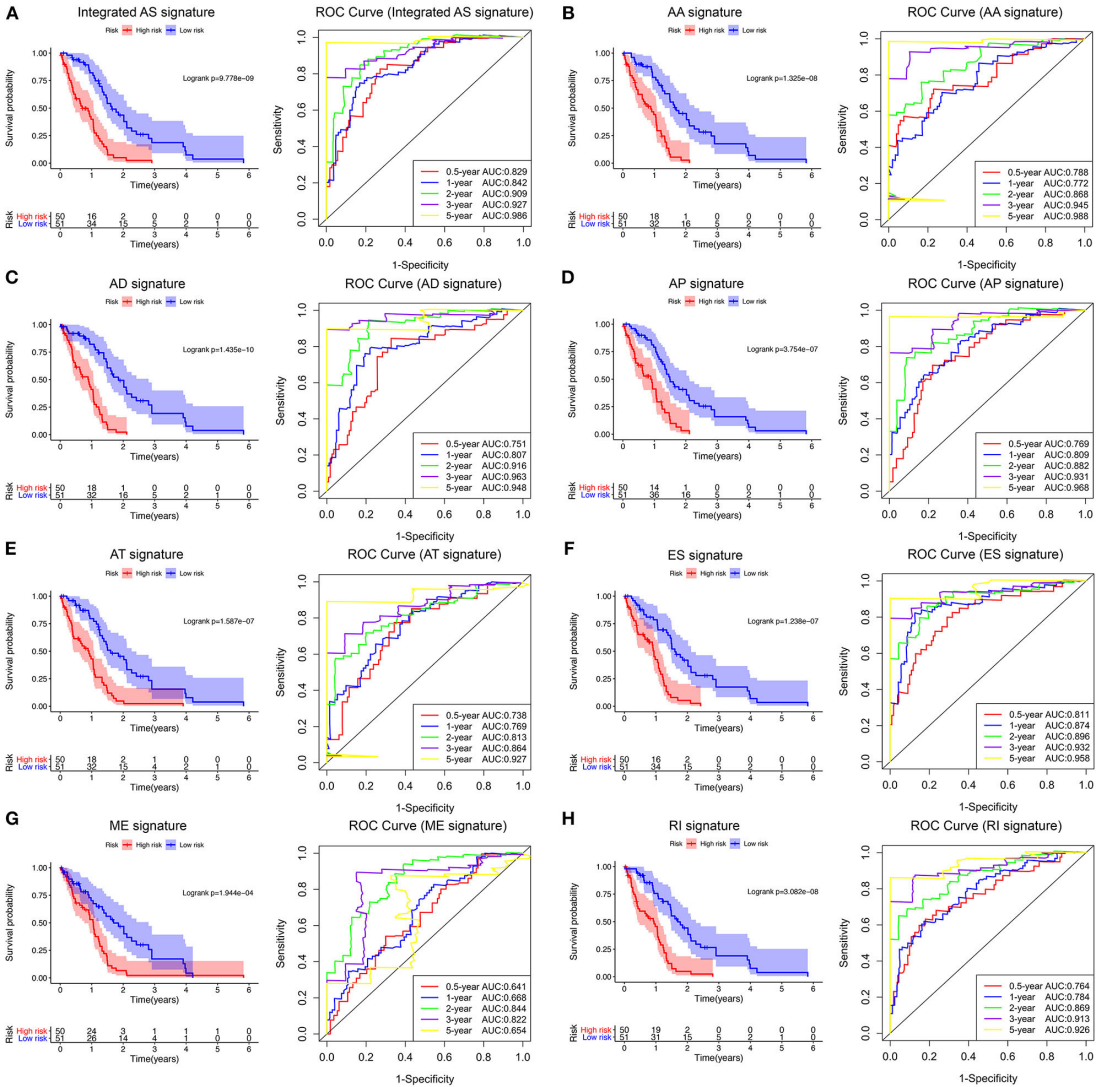
Survival analysis and prognostic performance of the integrated AS signature and the 7 type-specific AS signatures of GBM patients in the TCGA training set. Left panel **(A–H)** K-M survival curve of the risk score for the OS of GBM patients. The high-risk group had significantly poorer OS rates than the low-risk group. Right panel **(A–H)** The prognostic performance of the AS-based signature, demonstrated by the time-dependent ROC curves for predicting 0.5-, 1-, 2-, 3-, and 5-year OS in GBM patients.

Following the univariate Cox regression analysis, LASSO and multivariate Cox regression analysis were applied to all AS types combined and to each of the 7 AS types separately to screen the prognosis-associated AS events (Figure 3, Supplementary Table 1). ROC curves indicated excellent discriminative performances of LASSO analysis (Supplementary Figure 1). Eight prognostic risk score models based on AS events were established by the formulas shown in Table 2. Then, we calculated the prognostic risk score for each patient in the TCGA training cohort. The patients were divided into a high-risk group (high risk score) and a low-risk group (low risk score) using the median risk score as the cutoff (Figure 4). K-M survival curve analysis demonstrated that patients in high-risk groups had significantly poorer OS than patients in low-risk groups as defined by all eight types of AS signatures (log rank *P* <0.05; Figure 5). The C-indexes of the ES signature and the integrated AS signature for OS prediction were 0.875 (95% CI, 0.836–0.914; *P* = 6.05 × 10^−32^) and 0.852 (95% CI, 0.813–0.891; *P* = 5.99 × 10^−26^), respectively, demonstrating that these two models had the most favorable predictive value among the eight AS-based risk score models (Table 2). Furthermore, in time-dependent ROC analyses, all eight types of AS signatures showed favorable predictive ability for 0.5-, 1-, 2-, 3-, and 5-year OS, with each signature having an AUC of ~0.9 (Figure 5). Finally, to evaluate that the AS-based prognostic model had similar predictive performances in different populations, we applied it to predict OS in an independent internal validation cohort in a similar way. According to the risk score model, the 51 GBM patients in the validation cohort were divided into high-risk and low-risk groups (Supplementary Figure 2), and the OS of patients with high risk scores was significantly poorer than those with low risk scores in all eight types of AS signatures (logrank *P* < 0.05; Supplementary Figure 3). All eight types of AS signatures also showed favorable predictive abilities of the 0.5-, 1-, 2, 3-, and 5-year OS rates, with AUC of approximately 0.9, in the validation set (Supplementary Figure 3). These results indicate that all eight types of AS signatures may be robust and reliable prognostic predictors for GBM patients.

**Table 2:** The prognostic risk score models based on the PSI values of AS event types of GBM training cohort and C-index of the training and validation cohort.

**AS event types**	**Formula (risk score model)**	**Training set**		**Validation set**	
		**C-index (95%CI)**	***P*-value**	**C-index (95%CI)**	***P*-value**
Integrated AS signature	MAP3K13-68008-AA × (−9.16) + TMEM63B-76352-AP ×3.76 + MTF2-3772-ES × (−5.41) + ZNF302-48995-AD × (−2.27) + KLHL12-9424-ES × (−19.59) + ZNF280D-30765-AP ×4.72 + FAM86B1-82719-AD × (−2.59) + GSG1L-35696-AP × (−0.71) + PPAPDC1A-13279-AP × (−11.22) + HAT1-55964-ES × (−18.27)	0.852 (0.813–0.891)	5.99 ×10^−26^	0.843 (0.804–0.882)	2.78 ×10^−14^
AA signature	MAP3K13-68008-AA × (−5.38) + ATXN3-28923-AA × (−21.07) + BTBD10-14452-AA × (−12.90) + STAT3-41034-AA × (−17.42) + FAM193B-74803-AA × (−8.20) + METTL22-33899-AA × (−15.60) + EIF3B-78612-AA ×4.01 + PXK-65445-AA × (−3.03) + PROM2-54495-AA × (−3.65) + CHD4-19897-AA × (−3.78) + TK1-43785-AA × (−25.70) + FAM156B-89171-AA ×20.74	0.841 (0.802–0.880)	2.89 ×10^−19^	0.850 (0.812–0.889)	1.56 ×10^−11^
AD signature	ZNF302-48995-AD × (−4.21) + FAM86B1-82719-AD × (−2.94) + SERGEF-14562-AD ×1.83 + ZBTB45-52479-AD ×2.76 + SNX15-16731-AD × (−7.43) + C12orf57-20020-AD × (−17.45) + PACS2-29637-AD × (−11.97) + CFL2-27169-AD × (−27.55) + CHTF18-33022-AD × (−2.62) + SERPINB6-75151-AD ×3.66	0.828 (0.789–0.867)	2.74 ×10^−18^	0.859 (0.820–0.898)	1.92 ×10^−21^
AP signature	TMEM63B-76352-AP ×3.62 + ZNF280D-30765-AP ×4.64 + GSG1L-35696-AP × (−0.79) + PPAPDC1A-13279-AP × (−8.23) + TES-81522-AP × (−14.68) + RPL39L-68071-AP ×1.08 + DYRK3-9590-AP × (−10.01) + MEF2A-32714-AP ×2.56	0.824 (0.785–0.863)	7.83 ×10^−23^	0.805 (0.766–0.864)	3.05 ×10^−15^
AT signature	DST-76557-AT × (−14.41) + SYNE1-78181-AT × (−4.04) + CREBRF-74575-AT × (−14.26) + CCDC40-44016-AT ×3.03 + AIFM2-12029-AT × (−7.32) + OSBPL1A-44880-AT × (−9.95) + CDKL3-73367-AT ×2.83 + ECE2-67861-AT ×1.85 + CLCN5-89131-AT × (−9.06)	0.819 (0.780–0.858)	2.39 ×10^−21^	0.879 (0.839–0.918)	9.73 ×10^−11^
ES signature	MTF2-3772-ES × (−5.66) + KLHL12-9424-ES × (−23.17) + HAT1-55964-ES × (−15.71) + CBLL1-81372-ES ×3.20 + RWDD1-77328-ES × (−6.81) + R3HCC1L-12757-ES × (−20.61) + NKIRAS2-41007-ES × (−6.35) + UBXN11-101231-ES ×2.10 + FYN-124660-ES × (−3.10) + MTMR10-29794-ES × (−12.28) + SLC7A7-26626-ES × (−16.48)	0.875 (0.836–0.914)	6.05 ×10^−32^	0.867 (0.828–0.906)	4.71 ×10^−15^
ME signature	RPE-100824-ME × (−1.44) + FYN-77273-ME × (−2.74) + TTC13-10258-ME × (−1.26) + GRIA1-125279-ME ×0.88 + C4orf29-70560-ME × (−3.59) + C2CD5-251535-ME ×3.57	0.744 (0.705–0.783)	1.33 ×10^−6^	0.811 (0.772–0.850)	5.90 ×10^−19^
RI signature	SV2B-32540-RI × (−5.46) + TMEM170A-37612-RI × (−20.10) + COA6-10337-RI × (−2.60) + HOPX-69370-RI ×4.11 + MS4A6A-16057-RI × (−4.47) + UBC-25166-RI × (−6.38) + CRYAB-18698-RI × (−5.00) + LY6K-85358-RI × (−0.97) + GDA-86591-RI × (−5.67) + MRPL27-42373-RI × (−5.66) + C11orf49-15610-RI × (−2.29) + COX11-42567-RI ×1.19 + PRKRA-56163-RI × (−7.62)	0.833 (0.794–0.872)	5.53 ×10^−23^	0.834 (0.795–0.873)	3.80 ×10^−25^

*AS, alternative splicing; PSI, percent spliced in; GBM, glioblastoma; AA, alternate acceptor site; AD, alternate donor site; AP, alternate promoter; AT, alternate terminator; ES, exon skip; ME, mutually exclusive exons; RI, retained intron; CI, confidence interval*.

Correlation analysis of the eight AS signatures indicated that integrated AS, AA, and RI signature was significantly positively correlated with each other in both training (Supplementary Figure 4A) and validation cohort (Supplementary Figure 4B). In addition, the genes within the same signature did not show significant correlations (all correlation coefficient < |0.6|) in both training and validation set, which excluded colinearity among these genes (Supplementary Figures 4C–J).

### Evaluation of the Eight Types of AS Signatures as Independent Prognostic Factors

As shown in Table 3, univariate and multivariate Cox regression analyses were performed to evaluate the prognostic significance of the eight types of AS signatures together with various clinicopathological parameters. First, univariate analysis indicated that age (*P* < 0.001), new events (*P* = 0.003), pharmaceutical therapy (*P* < 0.001), radiation therapy (*P* = 0.001) and IDH mutation status (*P* = 0.009), integrated AS signature (*P* < 0.001), AA signature (*P* < 0.001), AD signature (*P* < 0.001), AP signature (*P* < 0.001), AT signature (*P* < 0.001), ES signature (*P* < 0.001), ME signature (*P* < 0.001), and RI signature (*P* < 0.001) were significantly associated with OS. Then, the multivariate analyses demonstrated that age (*P* = 0.010), new events (*P* < 0.001), pharmaceutical therapy (*P* = 0.011), radiation therapy (*P* = 0.018), AD signature (*P* < 0.001), and ES signature (*P* < 0.001) were significantly correlated with OS. Additionally, following the univariate and multivariate Cox regression analyses in the validation set, AD and ES signature were also proven to be independent prognostic predictors for GBM (Table 2). Interestingly, among the eight types of AS signatures, only AD and ES signatures were identified as independent prognostic factors for GBM.

**Table 3:** Univariate and multivariate cox proportional hazards analysis of clinical parameters and AS event-based risk score model of the TCGA GBM patients in the training cohort (*n* = 101) and internal validation cohort (*n* = 55).

**Variables**	**Training set (*****n*** **=** **101)**				**Validation set (*****n*** **=** **51)**			
	**Univariate Analysis**		**Multivariate analysis**		**Univariate Analysis**		**Multivariate analysis**	
	**HR (95%CI)**	***P***	**HR (95%CI)**	***P***	**HR (95%CI)**	***P***	**HR (95%CI)**	***P***
Age	1.028 (1.013–1.044)	** <0.001**	1.024 (1.006–1.043)	**0.010**	1.211 (1.171–1.249)	** <0.001**	1.088 (1.049–1.127)	** <0.001**
Sex	0.912 (0.623–1.334)	0.635	–	–	0.877 (0.485–1.269)	0.775	–	–
New event	0.568 (0.389–0.829)	**0.003**	0.439 (0.271–0.710)	**<0.001**	0.377 (0.338–0.416)	**<0.001**	0.347 (0.308–0.386)	**<0.001**
KPS	0.926 (0.695–1.233)	0.598	–	–	1.112 (0.721–1.504)	0.545	–	–
Pharmaceutical therapy	1.269 (1.129–1.425)	**<0.001**	1.114 (1.075–1.153)	**0.011**	1.215 (1.177–1.264)	**<0.001**	1.187 (1.168–1.226)	**0.032**
Radiation therapy	0.432 (0.262–0.712)	**0.001**	0.577 (0.366–0.908)	**0.018**	0.757 (0.618–0.796)	**0.005**	0.889 (0.944–0.929)	**0.045**
Surgery	0.962 (0.539–1.716)	0.895	–	–	0.911 (0.519–1.313)	0.798	–	–
IDH mutation status	0.263 (0.096–0.716)	**0.009**	1.261 (0.381–4.173)	0.704	0.853 (0.804–0.8922)	**0.021**	0.931 (0.5391–1.724)	0.334
Integrated AS signature (Low/High risk score)	3.604 (2.415–5.378)	**<0.001**	1.426 (0.856–2.376)	0.173	3.313 (2.921–3.705)	**<0.001**	1.267 (0.875–1.669)	0.088
AA signature (Low/High risk score)	3.653 (2.431–5.488)	**<0.001**	1.513 (0.932–2.455)	0.094	3.593 (2.201–4.985)	**<0.001**	1.115 (0.723–2.507)	0.214
AD signature (Low/High risk score)	4.305 (2.863–6.472)	**<0.001**	2.422 (1.491–3.935)	**<0.001**	5.312 (3.273–6.351)	**<0.001**	4.899 (3.507–6.291)	**<0.001**
AP signature (Low/High risk score)	3.430 (2.290–5.136)	**<0.001**	1.542 (0.923–2.578)	0.098	2.745 (2.453–3.137)	**<0.001**	1.561 (0.769–1.958)	0.313
AT signature (Low/High risk score)	3.244 (2.174–4.841)	**<0.001**	1.498 (0.909–2.470)	0.113	3.677 (2.285–4.169)	**<0.001**	1.855 (0.946–2.547)	0.093
ES signature (Low/High risk score)	5.145 (3.382–7.828)	**<0.001**	4.355 (2.517–7.534)	**<0.001**	6.213 (5.612–6.813)	**<0.001**	4.461 (3.867–5.061)	**<0.001**
ME signature (Low/High risk score)	2.122 (1.454–3.097)	**<0.001**	1.208 (0.774–1.887)	0.406	1.866 (1.267–2.478)	**<0.001**	1.211 (0.819–2.603)	0.322
RI signature (Low/High risk score)	4.092 (2.687–6.231)	**<0.001**	1.461 (0.881–2.425)	0.142	3.810 (3.418–4.202)	**<0.001**	1.259 (0.659–1.859)	0.078

*AS, alternative splicing; GBM, glioblastoma; AA, alternate acceptor site; AD, alternate donor site; AP, alternate promoter; AT, alternate terminator; ES, exon skip; ME, mutually exclusive exons; RI, retained intron; HR, hazard ratio; CI, confidence interval; NA, not available; KPS, Karnofsky performance score; CT, chemotherapy; TMT, targeted molecular therapy; HT, hormone therapy*.

*“New event” included progression and recurrence. “Others” in pharmaceutical therapy included CT + TMT + HT, CT + TMT + Immunotherapy, and CT + Immunotherapy*.

*All statistical tests were two-sided*.

*Bold type means P <0.05*.

### Construction and Validation of a Nomogram With AS Signatures

**Figure 6:**
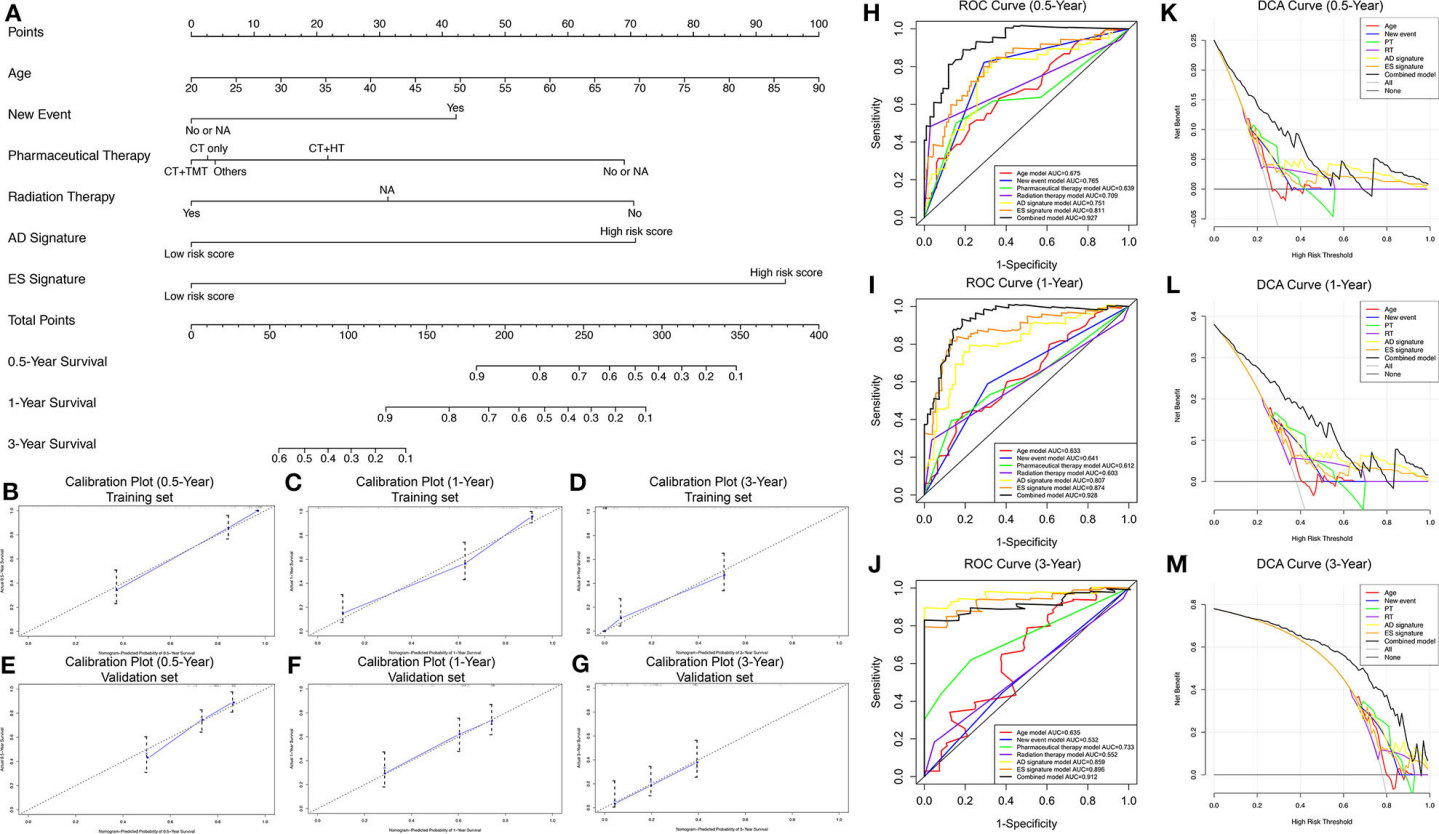
Nomogram to predict the probability of 0.5-, 1-, and 3-year survival in GBM. **(A)** Prognostic nomogram to predict the survival of GBM patients. **(B–D)** Calibration plots for the nomogram to predict survival at 0.5, 1, and 3 years in the training set. **(E–G)** Calibration plots for the nomogram to predict survival at 0.5, 1, and 3 years in the validation set. Actual survival is plotted on the y-axis, and the nomogram-predicted probability is plotted on the x-axis. **(H–J)** The prognostic performance of the nomogram, demonstrated by the ROC curves for predicting 0.5-, 1-, and 3-year OS, compared with other single factor prognostic models. **(K–M)** The clinical benefit and the scope of applications of the nomogram, evaluated by the DCA curves at 0.5, 1, and 3 years. The net benefit is plotted on the y-axis, and the threshold probabilities of patients for 1-, 3-, and 5-year survival are plotted on the x-axis.

To develop a clinically applicable model for predicting the prognosis of GBM, we constructed a nomogram to predict the probability of 0.5-, 1-, and 3-year survival of GBM patients. Six independent prognostic factors, including age, new events, pharmaceutical therapy, radiation therapy, AD signature and ES signature, were included in the prediction model (Figure 6A). The C-index of the nomogram was 0.892 (95% CI, 0.853–0.931; *P* = 5.13 ×10^−15^). The calibration plots (Figures 6B–D) achieved excellent agreement between the predicted and observed probabilities of 0.5-, 1-, and 3-year survival in GBM patients. The nomogram also showed powerful predictive ability for 0.5-, 1-, and 3-year OS, with AUC values of 0.927, 0.928, and 0.912, respectively (Figures 6H–J). As shown in Figures 6H–J, the discrimination performance of the nomogram was significantly higher than that of a prognostic model based on any of the six factors alone (age, new events, pharmaceutical therapy, radiation therapy, AD signature and ES signature). Additionally, DCA curves were applied to determine the clinical usefulness of the prognostic nomogram at 0.5, 1, and 3 years in GBM patients. As shown in Figures 6K–M, the nomogram demonstrated a greater net benefit than any of the single-factor prognostic models. In addition, in the TCGA internal validation cohort, the C-index of the nomogram for predicting survival of 51 GBM patients was 0.795 (95% CI, 0.756–0.834; *P* = 2.57 ×10^−10^). The calibration plots also indicated excellent agreements between survival prediction and actual observation in the probabilities of 0.5-, 1-, and 3-year OS in the validation cohort (Figures 3E–G). The nomogram achieved an AUC of 0.835, 0.738, and 0.776 for 0.5-, 1-, and 3-year OS, respectively, in the validation cohort (Supplementary Figure 5A). DCA curves also demonstrated a greater net benefit of the nomogram than other factors (Supplementary Figure 5B). Additionally, as shown in Supplementary Figure 6, the nomogram also achieved excellent predictive performances in both primary and recurrent GBM in the training cohort and validation cohort. These findings suggest that the nomogram is highly reliable in predicting the prognosis of GBM, meaning that it could assist both physicians and patients in performing individualized survival predictions and facilitate better treatment decision making and follow-up scheduling.

### Regulatory Networks Between Prognostic AS Events and SFs

**Figure 7:**
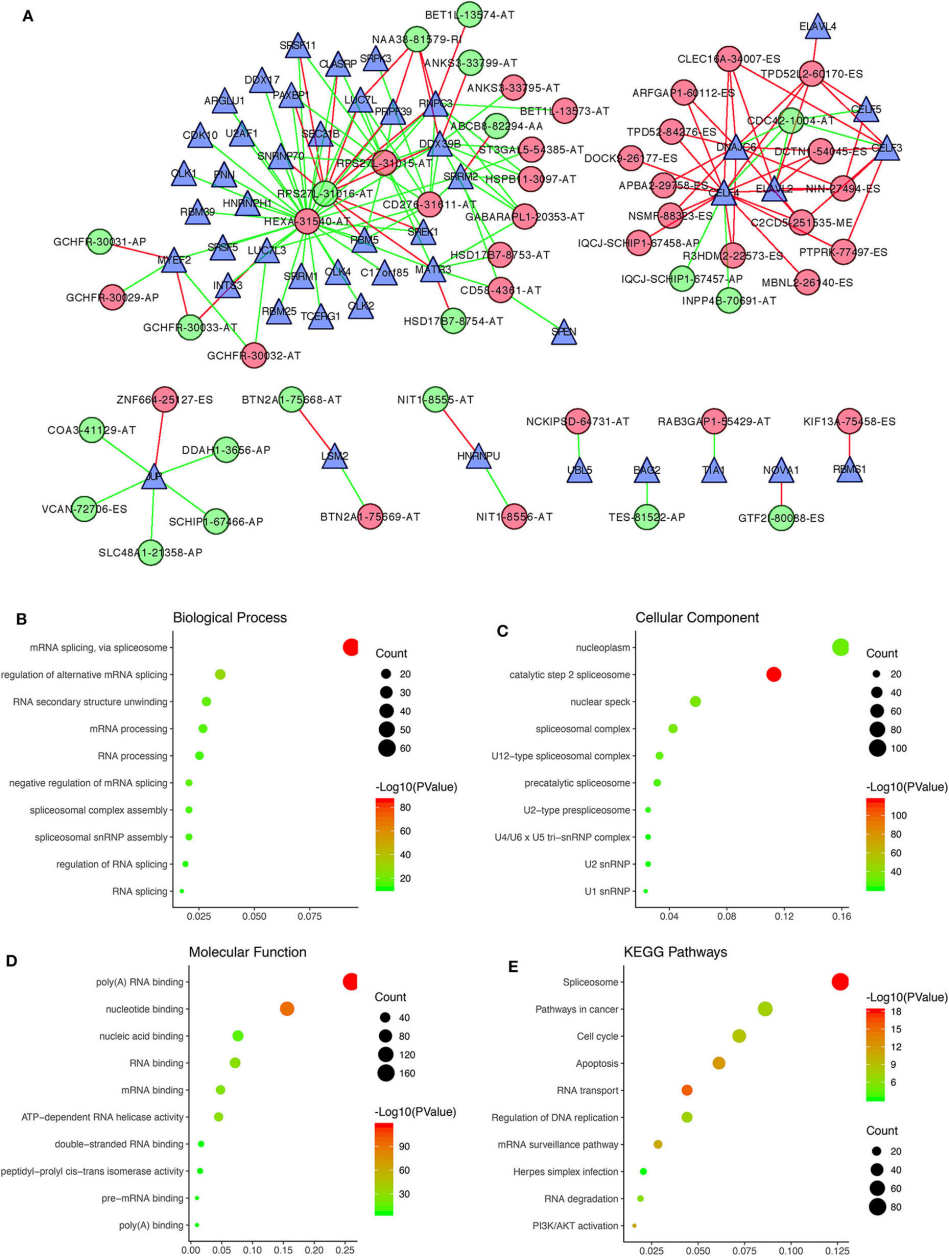
Correlation analysis between prognostic AS events and SFs. **(A)** The regulatory network between 47 survival-associated SFs and 52 survival-associated AS events, with |Pearson correlation coefficient| > 0.6 and *P* < 0.001. Green and red dots, respectively, represent favorable and unfavorable prognostic AS events. Green and red lines, respectively, represent negative and positive correlations between AS events and SFs. Blue triangles represent SFs. **(B–E)** Functional enrichment analysis of the 392 SF genes having |Pearson correlation coefficient| > 0.4 with prognostic AS events.

By performing survival analyses and correlation analyses of the RNA sequencing expression data combined with the AS sequencing data, we identified 47 survival-associated SFs and 52 survival-associated AS events that had significant correlations (Pearson correlation coefficient > 0.6 or < −0.6, *P* < 0.001; Supplementary Table 2). A total of 151 pairs of SFs-AS events, including 65 with positive correlations and 86 with negative correlations, were included in the regulatory network (Figure 7A). Interestingly, we found two subnetworks with different SF-AS correlations. In the subnetwork centered on HEXA-31540-AT, the majority of unfavorable prognostic AS events were negatively correlated with the expression of SFs, whereas the favorable prognostic AS events were positively correlated with the expression of SFs. In another subnetwork, centered on CELF4, the majority of unfavorable prognostic AS events were positively correlated with the expression of SFs, whereas the favorable prognostic AS events were negatively correlated with the expression of SFs.

Furthermore, functional enrichment analyses were performed for the 392 SF genes with |Pearson correlation coefficient| > 0.4. GO analysis, revealing that they were mainly enriched in mRNA splicing and regulation of alternative mRNA splicing within the BP category (Figure 7B), the nucleoplasm and the catalytic step 2 spliceosome within the CC category (Figure 7C), and poly(A) RNA binding and nucleotide binding within the MF category (Figure 7D). As for the KEGG pathways, the 392 SF genes were mainly enriched in spliceosomes, pathways involved in cancer, the cell cycle and apoptosis (Figure 7E).

## Discussion

AS is reported to be an important process modifying gene isoforms, which cause cells to produce different mRNA and protein isoforms with various functional properties in normal physiological processes (7, 8). Emerging evidence has demonstrated that dysregulated AS events play a vital role in the origin and progression of multiple cancers, especially GBM (9, 10). Aldave et al. (13) reported that the aberrant splicing regulation of BAF45d contributed to the malignant phenotype of GBM. Ferrarese et al. (29) demonstrated that lineage-specific splicing of a brain-enriched alternative exon promotes GBM progression. In addition, AS can serve as a therapeutic target for GBM. For instance, manipulating AS of the mRNA for the kinase Mnk2 (MKNK2) with splice-switching oligonucleotides (SSOs) was reported as a novel approach to inhibit glioblastoma tumorigenesis (12). In summary, numerous GBM-specific AS events and their mRNA isoforms have been identified, but there is still a lack of systematic analysis of survival-associated AS event profiles and prognostic prediction models based on multiple AS events for GBM.

In this study, we identified prognosis-related AS events and their source genes for the first time by performing univariate Cox regression analysis. A total of 1,598 (3.5%) AS events were associated with the survival of the TCGA GBM patients. Interestingly, more than half of the prognostic AS events were favorable prognostic factors. GO analysis and KEGG pathway enrichment analysis revealed that the prognostic source genes of the above AS events were mainly enriched in the pathways related to cancer and mRNA splicing. Then, following LASSO and multivariate Cox regression analysis, we constructed eight prognostic risk score models based on all AS types combined and each of the 7 AS types separately. All eight AS-based signatures showed excellent performance in distinguishing the survival of GBM patients. However, only two of them, the AD and ES signatures, were ultimately identified as independent prognostic factors for GBM compared with other clinicopathological parameters. Previous studies have investigated the novel prognostic value of various AS events and constructed the corresponding prognostic prediction models based on these AS events in multiple cancers, such as bladder urothelial carcinoma, renal clear cell carcinoma, and lung cancer (30–32). However, prognostic prediction models based on multiple AS events for GBM have not been reported before in the literature. Hence, the novel prognostic signature based on the AS events in our study, mainly AD and ES signatures, can be used for individualized survival predictions for GBM patients.

Nomogram models have been widely used in clinical practice due to its intuitive visual presentation (24). To the best of our knowledge, this is the first prognostic nomogram that incorporates AS-based signatures to predict the survival of GBM patients and was constructed from a large-scale database with long-term follow-up. In this study, we established a nomogram with age, new events, pharmaceutical therapy, radiation therapy, AD signature and ES signature. The calibration plots based on the TCGA GBM cohort demonstrated that the actual survival rates closely corresponded to the predictions, suggesting that the predictive performance of the nomogram was excellent. Following an evaluation of clinical usefulness by DCA, we concluded that our visualized scoring system is a reliable tool to aid physicians in making individualized treatment strategies and survival predictions, which could facilitate better treatment decision-making and follow-up scheduling.

Previous studies have demonstrated that SFs regulate oncogenic AS events by binding to splice-regulatory sequence elements of specific genes (28). In this study, we performed correlation analyses and constructed the regulatory networks between prognostic AS events and SFs to investigate the underlying regulatory mechanisms in GBM. Interestingly, we found two subnetworks with different SF-AS correlations. In the subnetwork centered on HEXA-31540-AT, the majority of unfavorable prognostic AS events were negatively correlated with the expression of SFs, whereas the favorable prognostic AS events were positively correlated with SFs. In another subnetwork, centered on CELF4, the majority of unfavorable prognostic AS events were positively correlated with SFs, whereas the favorable prognostic AS events were negatively correlated with SFs. Our study provides a novel understanding of AS patterns and their correlations with SFs in GBM, which may eventually help to elucidate the underlying roles of oncogenic AS events in the development of GBM.

This study has some limitations. First, the clinicopathological information downloaded from the TCGA GBM database was limited and incomplete. Detailed information on neuroimaging, the extent of resection, radiation therapy and chemotherapy was not included in the Cox regression model. Second, the prediction model was not further validated in the external GBM database containing AS sequencing data. Additional large-scale, multicenter prospective clinical trials are needed in the future.

In conclusion, by performing a global expression profile assessment, we developed a reliable AS-based risk score model for subgroup classification, risk stratification, prognosis prediction, and identification of potential therapeutic targets for GBM. Then, we successfully established a novel, promising prognostic nomogram that uses AS signatures and clinicopathological factors for individualized survival prediction to facilitate better treatment strategy and decision-making. Finally, the regulatory networks between prognostic AS events and SFs were constructed, which may eventually help to elucidate the underlying mechanisms of oncogenic AS events in the development and progression of GBM.

## Data Availability Statement

Publicly available datasets were analyzed in this study. This data can be found here: The level 3 RNA sequencing data and clinical information of 156 GBM patients were downloaded from The Cancer Genome Atlas (TCGA, https://portal.gdc.cancer.gov/) database. AS events in GBM and their percent-splice-in (PSI) values were obtained from the TCGA SpliceSeq data portal (https://bioinformatics.mdanderson.org/TCGASpliceSeq/).

## Author Contributions

LG, XG, and CF performed the data curation and analysis. KD and WL analyzed and interpreted the results. ZW and BX drafted and reviewed the manuscript. All authors contributed to the article and approved the submitted version.

## Conflict of Interest

The authors declare that the research was conducted in the absence of any commercial or financial relationships that could be construed as a potential conflict of interest.

## Abbreviations

Abbreviations: AS: alternative splicing
GBM: glioblastoma
TCGA: the cancer genome atlas
SF: splicing factor
C-index: Harrell's concordance index
ROC: receiver operating characteristic
AD: Alternate Donor site
ES: Exon Skip
OS: overall survival
PSI: Percent-Spliced-In
AP: Alternate Promoter
AT: Alternate Terminator
ME: Mutually Exclusive Exons
RI: Retained Intron
GO: Gene Ontology
KEGG: Kyoto Encyclopedia of Genes and Genomes
LASSO: least absolute shrinkage and selection operator
AIC: Akaike information criterion
BIC: Bayesian information criterion
K-M: Kaplan-Meier
AUC: area under the curve
KPS: Karnofsky performance score
DCA: decision curve analysis
HR: hazard ratio
CI: confidence interval
BP: biological process
CC: cellular component
MF: molecular function
MKNK2: manipulation of the kinase Mnk2
SSO: splice-switching oligonucleotides.
