# Supplementary figures and images for: Development of a Nomogram With Alternative Splicing Signatures for Predicting the Prognosis of Glioblastoma: A Study Based on Large-Scale Sequencing Data
Source: Front Oncol. 2020 Jul 22;10:1257. doi: 10.3389/fonc.2020.01257 (PMC7387698; doi:10.3389/fonc.2020.01257)

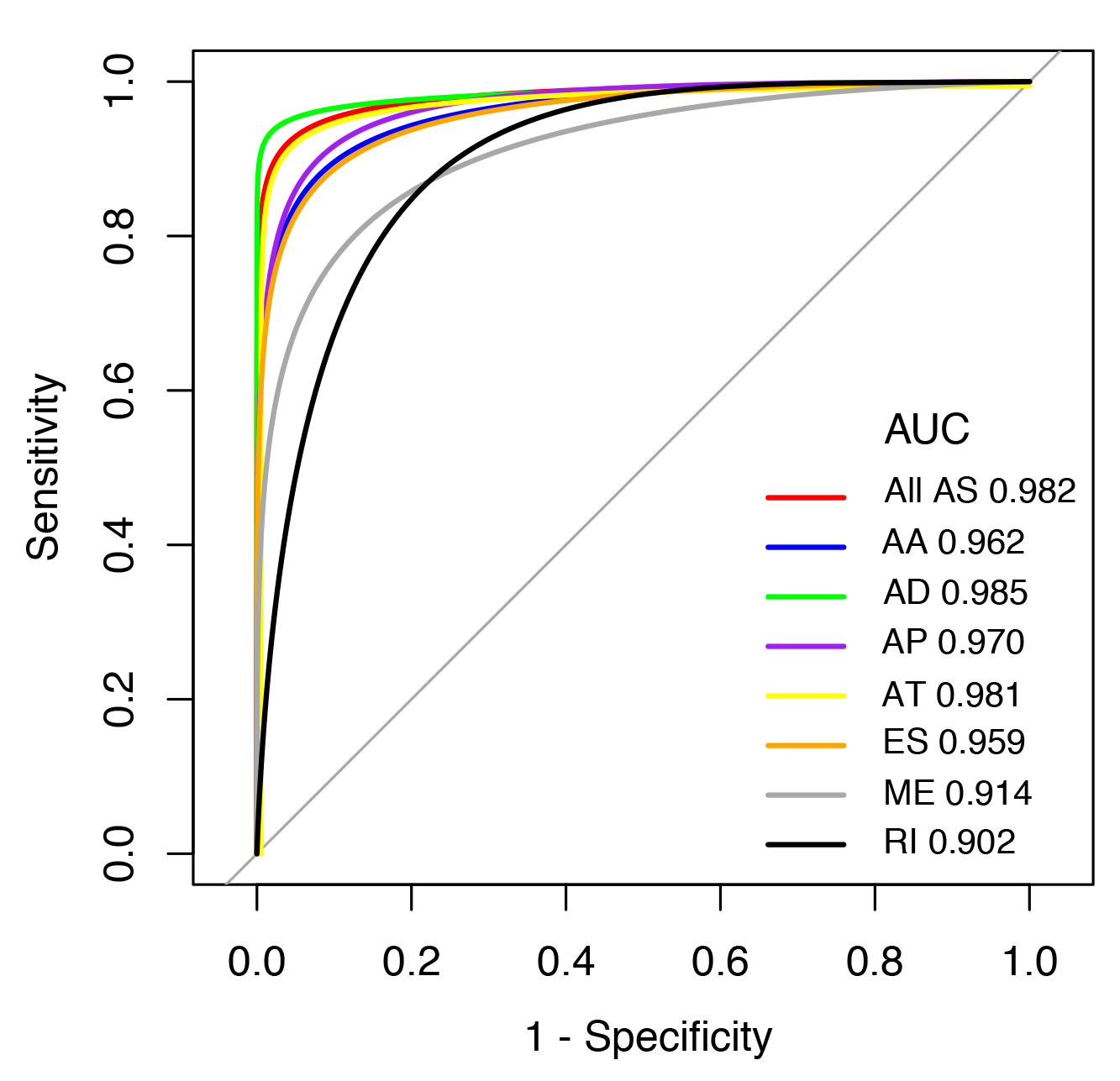

Supplement: Supplementary file 1 [file Image_1.TIF]

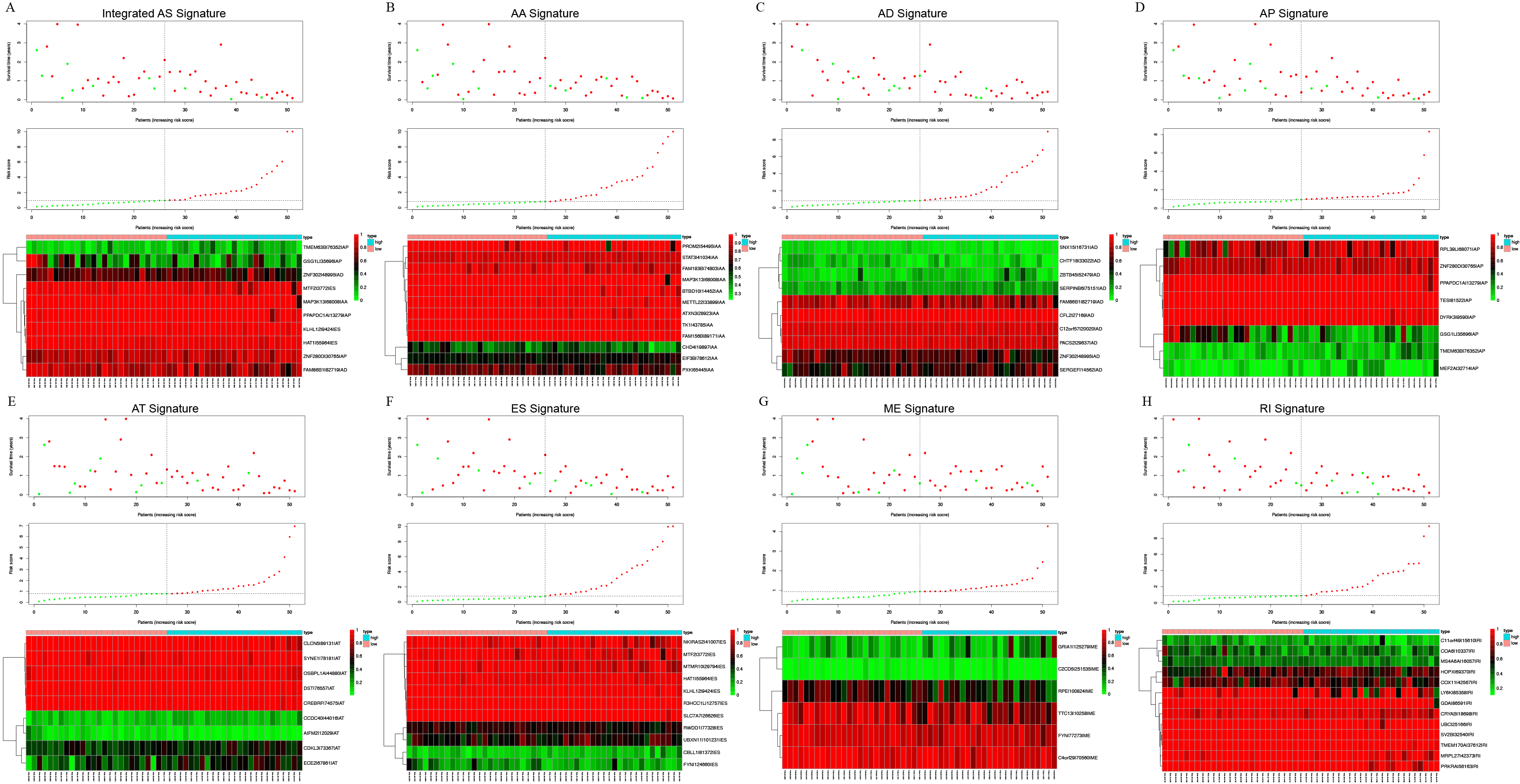

Supplement: Supplementary file 2 [file Image_2.TIF]

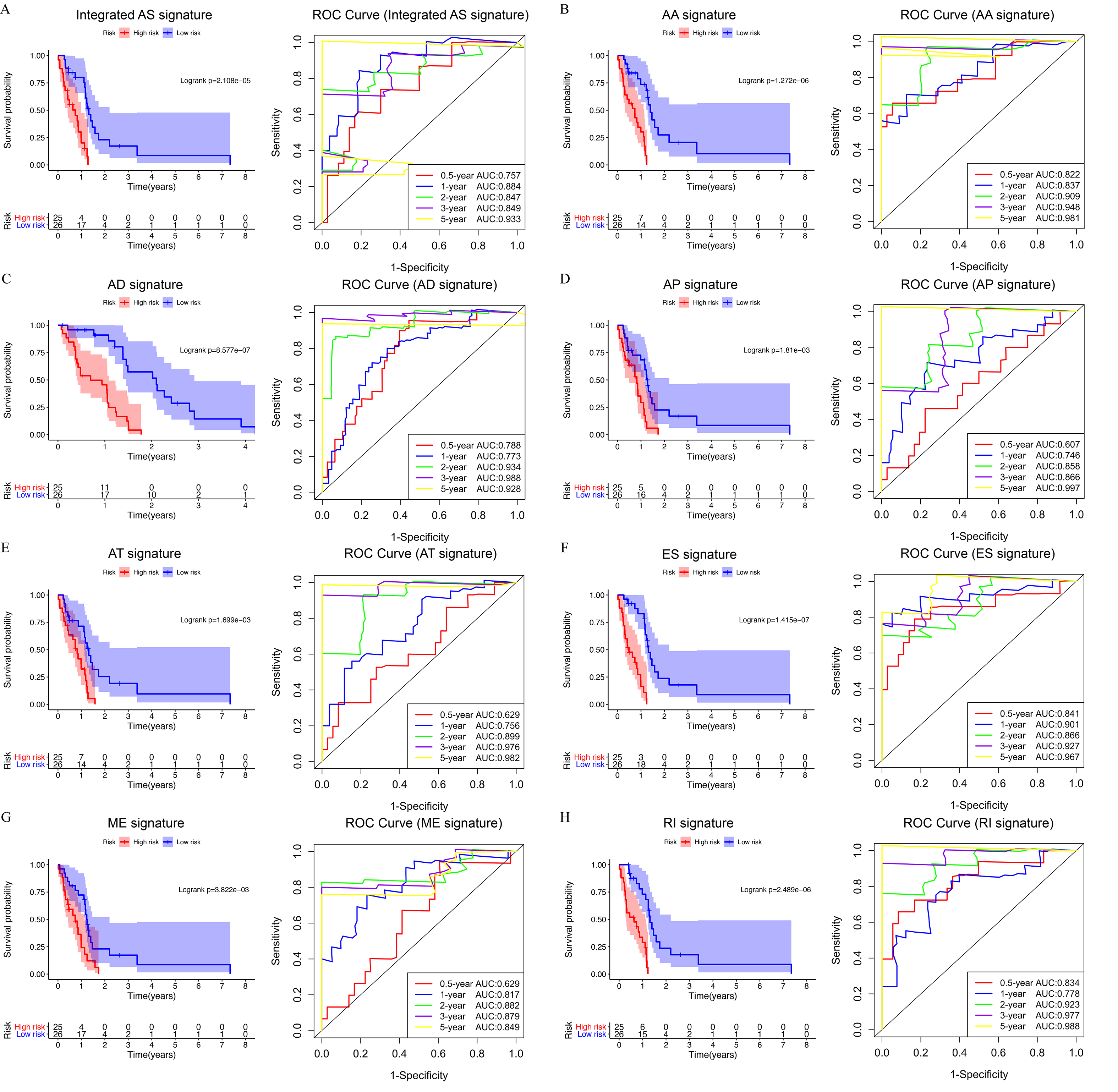

Supplement: Supplementary file 3 [file Image_3.TIF]

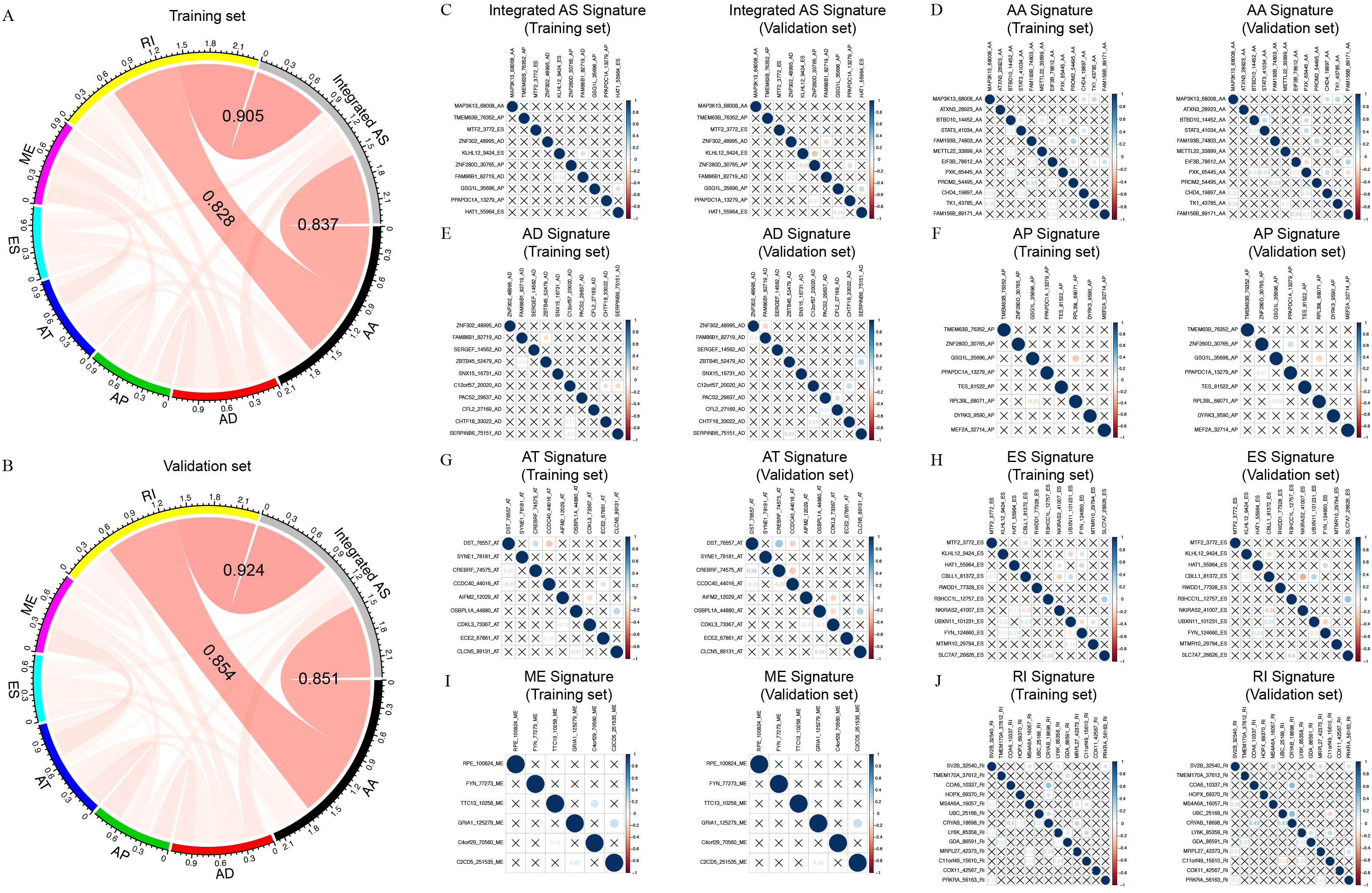

Supplement: Supplementary file 4 [file Image_4.TIF]

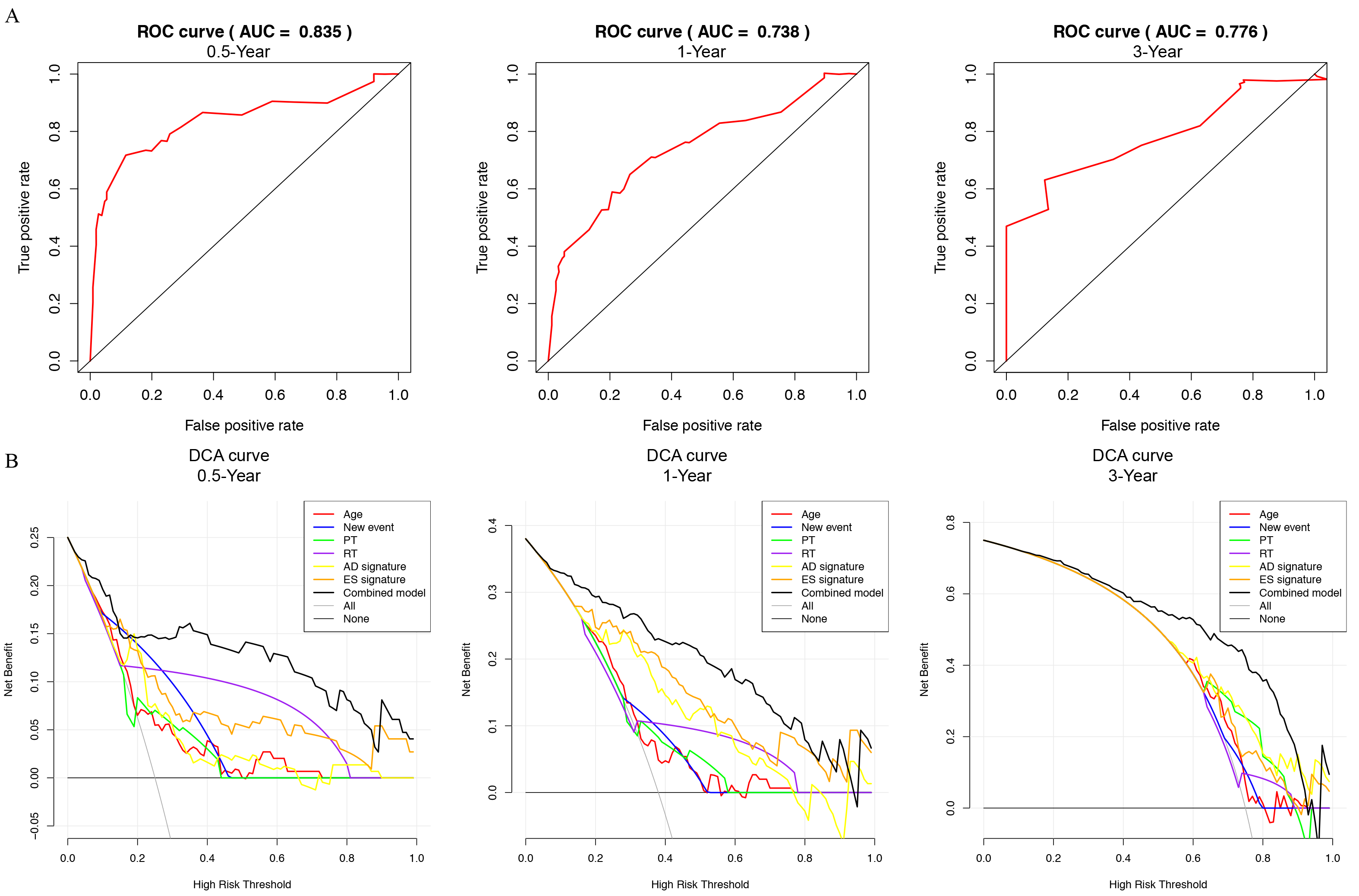

Supplement: Supplementary file 5 [file Image_5.TIF]

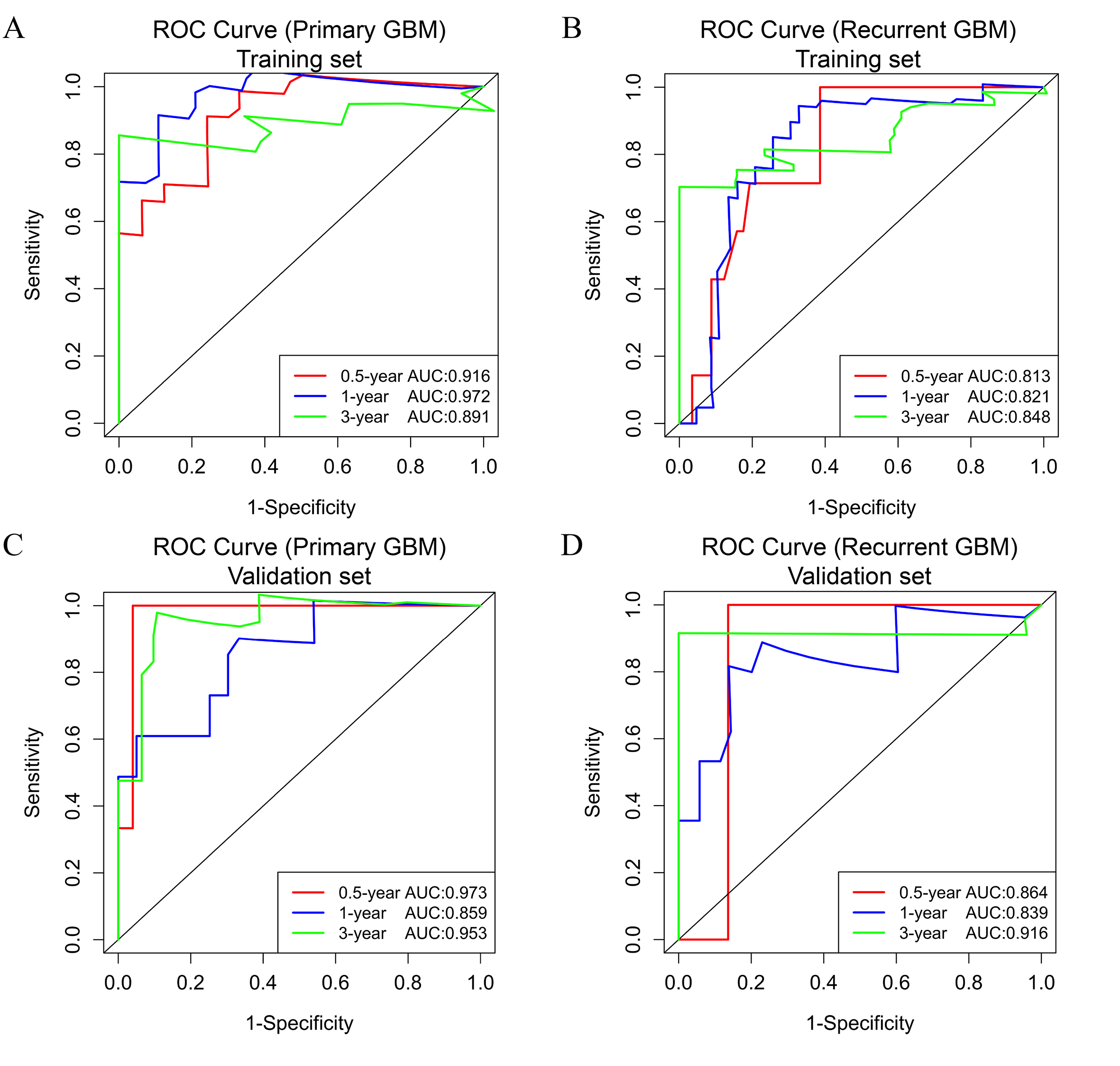

Supplement: Supplementary file 6 [file Image_6.TIF]
