# Supplementary material for: Development of a Nomogram With Alternative Splicing Signatures for Predicting the Prognosis of Glioblastoma: A Study Based on Large-Scale Sequencing Data
Source: Front Oncol. 2020 Jul 22;10:1257. doi: 10.3389/fonc.2020.01257 (PMC7387698; doi:10.3389/fonc.2020.01257)
Supplement: Supplementary file 9 [file Data_Sheet_1.docx]

# Supplementary figure legends

**Supplementary Figure 1.** The discriminative performances of LASSO regression analysis were demonstrated by the ROC curves.

**Supplementary Figure 2.** Risk score analysis of the integrated AS signature and the 7 type-specific AS signatures in the validation set. **Upper panel (A-H):** Patient survival status and time distributed by risk score. **Middle panel (A-H):** Risk score curve of the AS signature. **Bottom panel (A-H):** Heatmaps of prognosis-related AS events. Colors ranging from green to red indicate expression levels ranging from low to high. The dotted line represents the individual inflection point of the risk score curve, by which the patients were categorized into low-risk and high-risk groups.

**Supplementary Figure 3.** Survival analysis and prognostic performance of the integrated AS signature and the 7 type-specific AS signatures of GBM patients in the validation set. **Left panel (A-H):** K-M survival curve of the risk score for the OS of GBM patients. The high-risk group had significantly poorer OS rates than the low-risk group. **Right panel (A-H):** The prognostic performance of the AS-based signature, demonstrated by the time-dependent ROC curves for predicting 0.5-, 1-, 2-, 3-, and 5-year OS in GBM patients.

**Supplementary Figure 4.** Correlation analysis of the integrated AS signature and the 7 type-specific AS signatures in the training **(A)** and validation **(B)** set. The deep red means correlation coefficient > 0.6 and P < 0.05. **(C-J)** Correlation analysis of the genes within the same signature in the training and validation set. Blue stands for positive correlation, and red for negative correlation. The black cross stands for P > 0.05.

**Supplementary Figure 5.** **(A)** The prognostic performance of the nomogram, demonstrated by the ROC curves for predicting 0.5-, 1‐, and 3‐year OS in the validation cohort. **(B)** The clinical benefit and the scope of applications of the nomogram, evaluated by the DCA curves at 0.5, 1, and 3 years in the validation cohort. The net benefit is plotted on the y-axis, and the threshold probabilities of patients for 1‐, 3‐ and 5‐year survival are plotted on the x-axis.

**Supplementary Figure 6.** The prognostic performance of the nomogram, demonstrated by the ROC curves for predicting 0.5-, 1‐, and 3‐year OS of primary and recurrent GBM patients in the training cohort and validation cohort.
